# Menopause-related cortical loss of the humeral head region mainly occurred in the greater tuberosity

**DOI:** 10.3389/fendo.2022.942803

**Published:** 2022-08-24

**Authors:** Yeming Wang, Jian Li, Yutao Men, Wanfu Wei

**Affiliations:** ^1^ Department of Orthopedics, Tianjin Hospital, Tianjin University, Tianjin, China; ^2^ Department of Radiology, Tianjin Hospital, Tianjin University, Tianjin, China; ^3^ Tianjin Key Laboratory for Advanced Mechatronic System Design and Intelligent Control, School of Mechanical Engineering, Tianjin University of Technology, Tianjin, China; ^4^ National Demonstration Center for Experimental Mechanical and Electrical Engineering Education, Tianjin University of Technology, Tianjin, China

**Keywords:** cortical bone, humeral head, menopause, age, greater tuberosity

## Abstract

**Aims:**

Proximal humerus fractures are commonly observed in postmenopausal women. The goal of this study was to investigate menopause-related changes in cortical structure of the humeral head.

**Materials and methods:**

Clinical computed tomography (CT) scans of 75 healthy women spanning a wide range of ages (20–72 years) were analyzed. For each subject, cortical bone mapping (CBM) was applied to create a color three-dimensional (3D) thickness map for the proximal humerus. Nine regions of interest (ROIs) were defined in three walls of the humeral head. Cortical parameters, including the cortical thickness (CTh), cortical mass surface density (CM), and the endocortical trabecular density (ECTD), were measured.

**Results:**

Compared to premenopausal women, postmenopausal women were characterized by a significantly lower CTh and CM value in the lateral part of the greater tuberosity. Similar changes were only found in ROI 4, but not in ROIs 5–6 in the lesser tuberosity. Linear regression analysis revealed that the CTh and CM value of ROIs 1, 3, and 4 were negatively associated with age. These results showed that menopause-related loss in CTh and CM was mainly in the greater tuberosity besides the proximal part of the lesser tuberosity. Trabecular bone variable measured as ECTD showed a notably lower value in ROIs 1–9 in postmenopausal vs. premenopausal group. Inverse linear associations for ECTD and age were found in ROIs 2, 3, 5, 6, 7, and 9, indicating no site-specific differences of endocortical trabecular bone loss between the greater and lesser tuberosity.

**Conclusions:**

Menopause-related cortical loss of the humeral head mainly occurred in the lateral part of the greater tuberosity. The increased rate of humeral bone loss in the greater tuberosity may contribute materially to complex proximal humerus fractures.

## Introduction

Proximal humerus fractures (PHFs) are common fragility fractures in elderly patients, second only to vertebral and hip fractures in terms of incidence (1). These fractures are associated with low bone mineral density (BMD) and increase in incidence after the age of 50 (1–3). Most PHFs are observed in postmenopausal women (3). Estrogen deficiency after menopause resulted in an unbalanced coupling between resorption and formation in favor of bone resorption, gradually producing microstructural deterioration and reduction of the mineral content of the bone material. Previous studies have concentrated on age-related changes of trabecular microstructure for its distinct remodeling (4, 5). However, cortical bone constitutes 80% of skeletal mineralized bone volume in adults, particularly at appendicular sites where the cortex accounts for the majority of axial load transfer (6, 7). Recent studies on the radius, femur, and humerus had found that bone loss during aging is predominantly cortical in origin and reaches a maximum around the age of 65 years (8, 9). Cortical bone accounted for over 80% of all the bone loss during and after menopause. Porosity increased in the compact-appearing, outer, and inner transitional zones of the cortex (10). In a 3-year prospective study using high-resolution peripheral QCT (HR-pQCT), an increase in endosteal perimeter and cortical porosity at the radius was detected in postmenopausal women, which partly led to an annual decline in the estimated failure load (11). Therefore, cortical loss has a more negative effect on mechanical stability than trabecular bone loss and contributes to skeletal fragility (8–12).

Bone strength is determined not only by bone mass but also by bone morphology as size, shape, and three-dimensional (3D) architecture and microarchitecture. The most important risk factor for bone loss in midlife women is menopause. The increases in the outer diameter of the femoral neck were found to parallel the reduction in BMD and section modulus during the menopause transition (13). These suggest that changes in bone size could contribute to an increased fracture risk, although they may partially compensate for bone loss resulting from endosteal resorption. Several cohort studies demonstrated that deficits in cortical and trabecular bone density and microstructure predict incident fracture independently of femoral neck BMD and FRAX (Fracture Risk Assessment Tool) score (14–16). Cortical BMD, thickness, and area at the tibia were considered as part of the best set of fracture predictors in these studies that can be expected, as the structural properties of cortical bone are proposed to be the major contributors to bone strength (14, 16, 17).

The proximal humerus is relatively under investigation as one of the most common sites of osteoporotic fracture. Few studies have explored age-related changes in trabecular bone properties at the proximal humerus (4, 6, 18). Little data are available for menopause-related changes of the cortical structure in the proximal humerus. The purpose of the present study was to evaluate the cortical bone characteristics of the proximal humerus in quantitative CT data obtained in healthy women before and after menopause.

## Materials and methods

### Subjects and study design

Individuals were participants in the aging and osteoporotic PHF study, a single-center prospective ongoing population study of Chinese men and women. Our analytical sample included 75 healthy women, aiming to evaluate menopause-related changes in cortical bone of the humeral head region in the dominant upper extremity. All subjects were Han Chinese. Menopause was defined as the date of the last menses followed by 12 months without menses. Thirty-five (46.7%) women were premenopausal, with a regular cycle in the last 3 months, and 40 (53.3%) were postmenopausal. Subjects with a history of or evidence of metabolic bone disease and those receiving chronic treatment that may affect bone metabolism were excluded from the study. Arm dominance was determined as the arm with which subjects would throw a ball. For this study, no dual-energy X-ray absorptiometry screening was performed prior to enrollment; therefore, no BMD inclusion/exclusion criteria were used. Written informed consent was obtained from all participants, and the study was approved by the institutional review board of Tianjin Hospital.

### Cortical bone mapping

CT scanning (Mx 8000 IDT; Philips Medical Systems, Best, Netherlands) was performed at 120 kV (peak) and 168 milliampere-seconds. CT images were created in slice increments of 2.00 mm at a resolution of 0.566 mm × 0.566 mm/pixel with a field of view of 29 cm × 29 cm. Subjects were positioned supine with their arms in neutral position and centered within the gantry of the machine. Each image was analyzed from the slice that included the top of the acromion to the slice that included the inferior angle of the scapula. All CT scanning was performed by JL. CT values of pixels were recorded in Hounsfield units (HUs).

The cortical parameter measurement and mapping technique have been previously described (19, 20). Cortical thickness (CTh) measurement was performed using cortical bone mapping (CBM), implemented by a freely available in-house program called Stradwin (http://mi.eng.cam.ac.uk/~rwp/stradwin/). First, an approximate segmentation of each proximal humerus from the CT data was performed using Stradwin and results in a triangulated surface mesh with ~10 (4) vertices distributed uniformly over the proximal humerus surface. Second, the CT data were sampled at each vertex of the mesh using 18-mm lines perpendicular to and passing through the humeral cortex and trabeculae. Finally, a model that accounts for the imaging blur was fitted to the data samples. This validated model-based deconvolution process allows the measurement of much smaller features than would normally be visible in the CT data. This process was repeated at all vertices. As a result, color maps on the proximal humerus were created for accurately estimating the CTh (in mm) and cortical mass surface density (CM, the cortical mass per unit surface area), as well as the endocortical trabecular density (ECTD), which is the trabecular density directly adjacent to the cortex.

### Definition of the regions of interest for cortical bone distribution assessment

**Figure 1:**
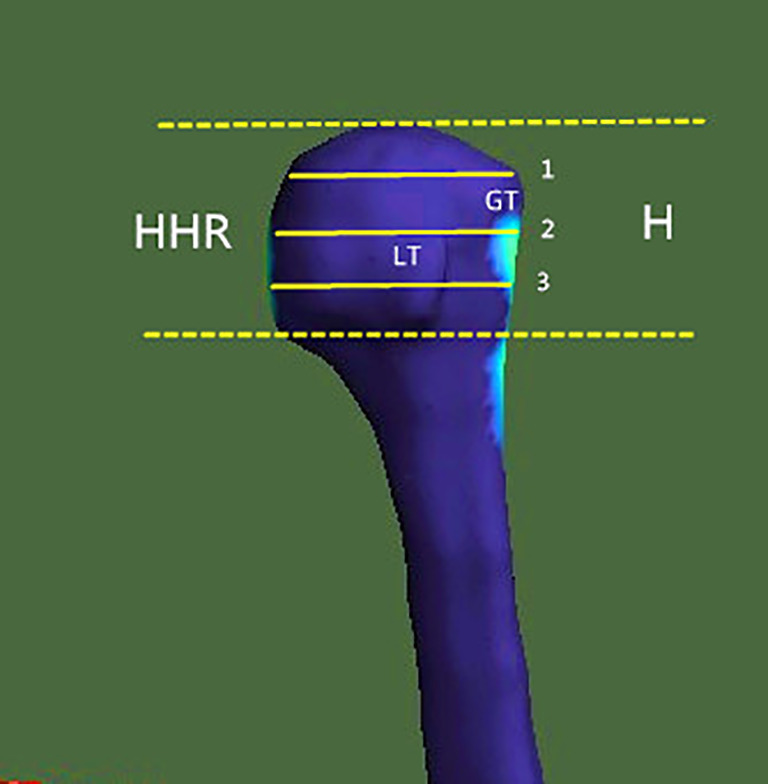
Region of investigation. The humeral head height (H) was the distance between the highest point of the humeral head and the most distal margin of the articular surface. In the humeral head region (HHR), cortical parameters were determined within different trisections of humeral head height. GT, greater tuberosity; LT, lesser tuberosity.

**Figure 2:**
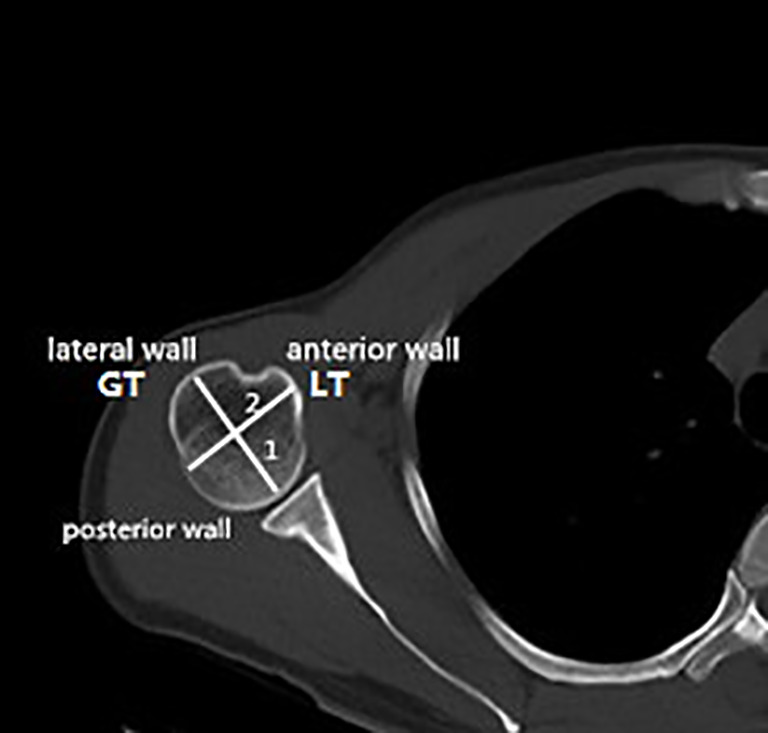
Locations of the measuring points in the humeral head region. Line 1, longest diameter between the articular surface and the greater tuberosity. Line 2, vertical bisection of line 1. GT, greater tuberosity; LT, lesser tuberosity.

**Figure 3:**
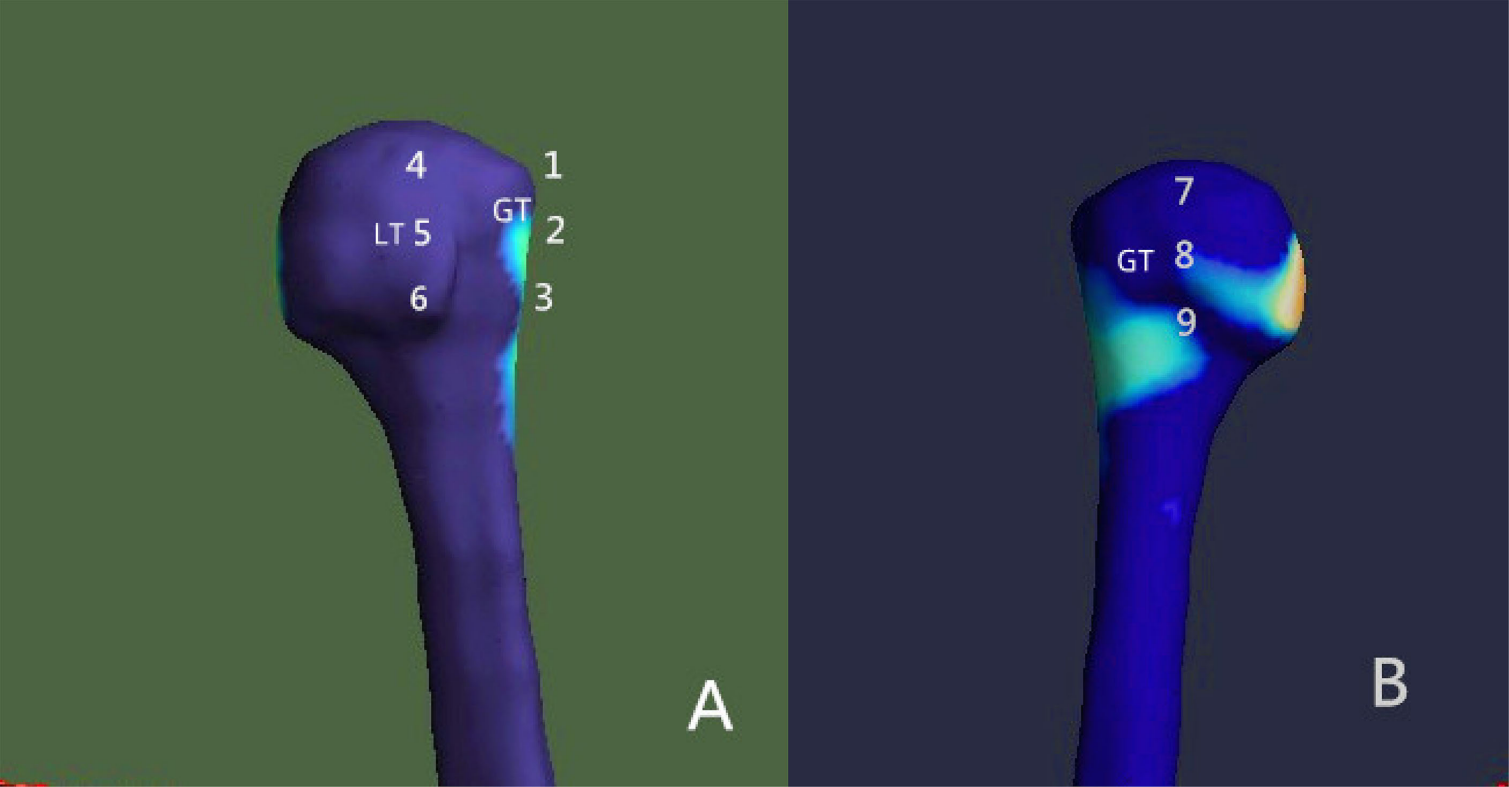
Placement of the regions of interest (ROIs) Nine ROIs were defined in the humeral head region. **(A)** Anterior view; **(B)** posterior view. GT, greater tuberosity; LT, lesser tuberosity.

For the evaluation of the bone morphometric analysis, specific regions of interest (ROIs) were defined within the proximal end of the humerus. The specific methodology has been described in detail previously and will be briefly outlined here (18). The cortical bone in the humeral head region was defined as anterior, lateral, and posterior walls. In an anatomical perspective, the anterior wall is equivalent to the lesser tuberosity. The lateral and posterior parts of the greater tuberosity correspond to the lateral and posterior walls. Following the creation of a single 3D thickness map, the humeral head height (H) was determined by measuring the distance between the highest point of the humeral head and the most distal margin of the articular surface (**Figure 1**). The height of the humeral head was then quartered by axial planes 1–3 that were equidistant to each other. In each slice, to obtain more details of cortical bone tissue, the longest line (Line 1) between the joint surface and greater tuberosity was drawn; this line was divided into a medial and a lateral segment by line 2, which intersected it at right angles (**Figure 2**). ROIs 1–9 were established as cortical bone measurement points (**Figure 3**).

### Statistical analysis

The cortical difference between premenopausal vs. postmenopausal group was compared using t tests for normally distributed values and Kruskal–Wallis test for non-normally distributed values. The correlation between cortical indices and age in ROIs 1–9 was studied by linear regression analysis. All statistical analyses were performed using IBM SPSS Statistics for Windows version 20.0 (IBM SPSS Inc., Chicago, IL, USA). Significance level was set at P < 0.05 for all statistical tests.

## Results

### Changes in morphology prior to and after menopause

The median age of the premenopausal and postmenopausal groups was 35 years (interquartile range, 27–43 years) and 65 years (interquartile range, 61–67 years), respectively. When compared to the premenopausal women, postmenopausal women were characterized by a significantly lower CTh and CM value of ROIs 1–3 in the lateral part of the greater tuberosity (all P < 0.05). Similar changes were only found in ROI 4 (all P < 0.05) but not in ROIs 5–6 in the anterior wall. In the posterior wall, no difference was detected between the two groups for either CTh or CM. These results indicated that menopause-related loss in CTh and CM was mainly in the greater tuberosity, but also the proximal part of the lesser tuberosity. Trabecular bone parameter measured as ECTD showed a notably lower value in ROIs 1–9 in the postmenopausal group, showing that endocortical trabecular loss occurred in both the greater and lesser tuberosity (all P < 0.05, **Table 1**).

**Table 1:** Menopause-related difference in variables of ROIs of the subjects.

Variables	Premenopausal(n=35)	Postmenopausal(n=40)	p value
Age CTh(mm)	34.83±9.13	64.80±6.77	
ROI1	5.24±1.28	3.37±1.74	0.00
ROI2	3.31±1.36	2.57±1.51	0.03
ROI3	3.23±1.28	2.34±1.06	0.02
ROI4	4.49±1.44	3.67±1.58	0.02
ROI5	4.10±1.49	3.52±1.71	0.12
ROI6	4.21±1.50	3.56±1.80	0.09
ROI7	2.77±0.99	2.18±0.90	0.84
ROI8	2.34±0.74	2.18±0.91	0.41
ROI9	2.97±1.28	2.79±1.71	0.53
CM(HUmm)			
ROI1	56401.68±17832.52	38621.11±22082.35	0.00
ROI2	45311.95±44759.36	29414.84±17783.92	0.04
ROI3	37257.85±17071.22	26409.81±11929.56	0.03
ROI4	49615.49±18884.85	40749.49±18470.29	0.04
ROI5	46744.91±18571.47	38875.54±19476.03	0.08
ROI6	48107.64±18513.44	40049.46±20818.40	0.08
ROI7	32088.01±13076.91	31503.35±15719.24	0.86
ROI8	26173.71±9264.46	24712.46±10513.65	0.53
ROI9	34894.03±16476.05	32391.99±14546.94	0.49
ECTD(HU)			
ROI1	10112.27±52.88	10015.68±228.10	0.01
ROI2	10085.80±46.49	9975.02±220.37	0.00
ROI3	10066.51±55.49	9953.70±222.96	0.01
ROI4	10140.62±198.61	10025.55±222.67	0.02
ROI5	10110.30±59.89	9995.32±216.77	0.00
ROI6	10122.42±72.51	9996.68±220.12	0.00
ROI7	10129.84±68.45	10020.52±210.33	0.00
ROI8	10097.12±65.95	10000.30±225.45	0.02
ROI9	10092.00±63.28	9972.49±227.40	0.00

The values are given as the mean and the standard deviation. CTh, cortical thickness, CM, cortical mass surface density, ECTD, the endocortical trabecular density.

### Age-related differences in cortical bone quality

**Figure 4:**
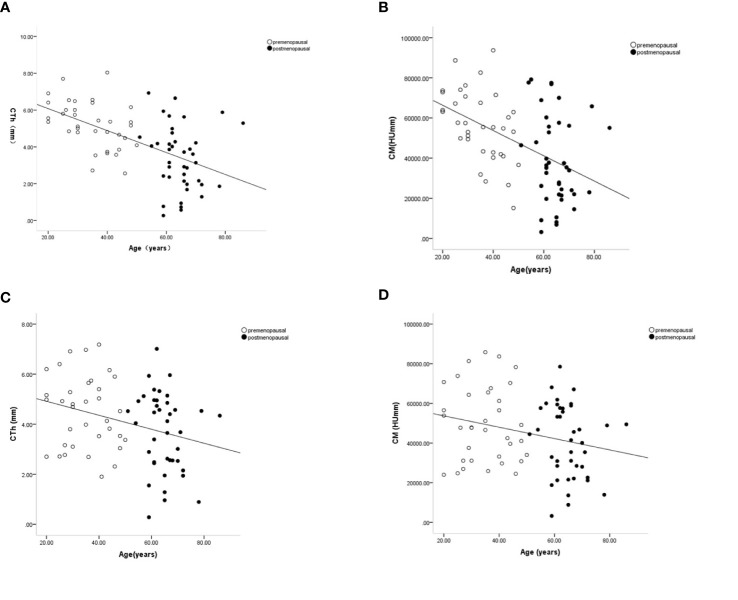
Age-related changes in cortical thickness and cortical mass surface density in the proximal part of the lateral wall (ROI 1) **(A, B)** and anterior wall (ROI 4) **(C, D)**. Premenopausal outcomes are indicated by open symbols, postmenopausal outcomes by full symbols. Solid lines represent the fitted mean from the regression models.

When pooled across all decades, linear regression analysis revealed that the CTh and CM values of ROIs 1, 3, and 4 were negatively associated with age (all P < 0.05) (**Figure 4**). Similarly, inverse linear associations for ECTD and age were found in ROIs 2, 3, 5, 6, 7, and 9 (all P < 0.05). It can be seen that the decline of CTh and CM with age occurred in the proximal part of the greater and lesser tuberosity, whereas there was no site-specific difference in endocortical trabecular bone loss between the greater and lesser tuberosity.

## Discussion

This study investigated the menopause-related changes in CTh, CM, and ECTD in specific regions of the humeral head region measured in a Chinese cohort by CBM technique. Our principal findings are as follows: 1) The predominant cortical loss occurred in the lateral part of the greater tuberosity after menopause; 2) Obvious cortical loss in the proximal parts of the greater and lesser tuberosity was detected in postmenopausal women; 3) The greater and lesser tuberosity had similar patterns of endocortical trabecular bone loss with aging.

Cortical bone bears the bulk of axial loads in the proximal humerus, and the distribution of the cortex is an important factor in bone strength and fracture prediction (18, 21). Our data demonstrated a main accentuation of cortical bone in the lateral part of the greater tuberosity after menopause. Meanwhile, ECTD decreased obviously in each ROI in the greater and lesser tuberosity, suggesting that excess endocortical resorption in postmenopausal women agreed with earlier histomorphometric analysis (4). The marked decrease of cortical bone thickness and mass surface density in the greater tuberosity indicated a structural weakness, which was closely connected with fracture for stress concentration effects. Focal cortical thinning in the greater tuberosity may play a vital role in proximal humerus fractures associated with falls. Previous studies had focused on spatial differences in proximal humeral CTh and discovered that proximal humerus fractures occur along lines of cortical thinning (22, 23). Furthermore, the isolated greater tuberosity fractures are believed to represent the commencement of a cascade of events that ultimately culminate in a shield-type proximal humerus fracture (23). Our finding might illuminate why complex proximal humerus fractures tend to initiate in a particular zone.

**Figure 5:**
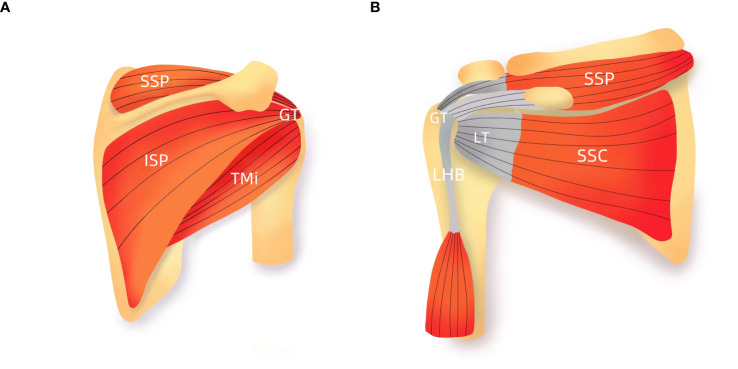
Schematic presentation of rotator cuff **(A)** Anterior view; **(B)** posterior view. GT, greater tuberosity; LT, lesser tuberosity; SSC, subscapularis; SSP, supraspinatus; ISP, infraspinatus; TMi, teres minor; LHB, the long head biceps tendon.

The gross properties of cortical bone change substantially after menopause. However, the pattern and magnitude of bone loss differ at various skeletal sites and may be related to local biomechanical load or to various degrees of response to decreased estrogen (11, 24). In normal gait, the greatest stresses occur in the subcapital and medial midfemoral neck regions, where maximum compressive stresses occur inferiorly (14). Superiorly, smaller-magnitude tensile stresses occur during walking. Accordingly, bone decrement occurs preferentially in the superior region than in the inferior region of the femoral neck during aging (14, 24). In this study, we found that the CTh of the proximal parts of the lateral and anterior walls of the humerus was lower significantly in postmenopausal women and negatively associated with age. Anatomically, the rotator cuff is attached to both the greater tuberosity and lesser tuberosity (**Figure 5**). The intrinsic properties of the proximal humerus cortex depend on mechanical loading from the rotator cuff activity, unlike the weight-bearing bones as proximal femur or tibia (21). We speculated that normal daily loading from the rotator cuff cannot prevent menopause and/or age-related cortical loss from the proximal part of the anterior and lateral walls of the humeral head. Consistent with our findings, Shanbhogue et al. (11) observed trabecular separation at the radius but not the tibia with advancing age and during the menopause transition. Taken together, we believed that it is possible that the humerus, as a non–weight-bearing bone, may have a higher sensitivity to decline in bioavailable estrogen levels leading to the observed bone loss.

Our study has several limitations. The most obvious limitation is the cross-sectional nature of the study that limits the ability to reflect age-related changes in bone geometry. Direct comparison of each cortical bone index in the premenopausal and postmenopausal groups could not distinguish between age-related and menopause-related effects. A longitudinal cohort study of women is needed to examine changes in proximal humeral bone health across the menopausal transition. Second, we have evaluated menopause-related cortical bone effects in a Chinese cohort. The current data are not directly translatable to individuals of other racial or ethnic backgrounds since previous work suggests structural differences of the proximal femur between Asians and other ethnicities (11, 24). Finally, microarchitectural changes of the cortical bone in the humeral head region were not analyzed in the study. Some authors recently reported that cortical porosity and thickness have a significant impact on bone loss and mechanical stability (8, 9, 11). Despite this limitation, we identified menopause-related changes in cortical bone of the humeral head region, which are definitely relevant to risk prediction for PHFs.

In summary, we have shown that menopause-related cortical loss of the humeral head mainly occurred in the lateral part of the greater tuberosity. Since fractures initiate from focal cortical thinning, the increased cortical bone loss in the greater tuberosity may contribute materially to complex PHFs. CTh in the proximal part of the lateral and anterior walls exhibited significant age- and menopause-related decline in women. Collectively, cortical loss in the greater tuberosity and the lesser tuberosity showed marked regional heterogeneity under the impact of estrogen deficiency and/or aging. Better understanding of the mechanisms determining local bone loss in elderly proximal humerus is an important topic for future research.

## Data availability statement

The raw data supporting the conclusions of this article will be made available by the authors, without undue reservation.

## Ethics statement

This study was reviewed and approved by The ethics committee of Tianjin Hospital. The patients/participants provided their written informed consent to participate in this study. Written informed consent was obtained from the individual(s) for the publication of any potentially identifiable images or data included in this article.

## Author contributions

YW designed the study and prepared the first draft of the paper. JL and YM contributed to the experimental work. WW was responsible for project Administration. All authors revised the paper critically for intellectual content and approved the final version. All authors agree to be accountable for the work and to ensure that any questions relating to the accuracy and integrity of the paper are investigated and properly resolved.

## Funding

This study was supported by Tianjin National Science Foundation of China (18JCYBJC95200).

## Conflict of interest

The authors declare that the research was conducted in the absence of any commercial or financial relationships that could be construed as a potential conflict of interest.

## Publisher’s note

All claims expressed in this article are solely those of the authors and do not necessarily represent those of their affiliated organizations, or those of the publisher, the editors and the reviewers. Any product that may be evaluated in this article, or claim that may be made by its manufacturer, is not guaranteed or endorsed by the publisher.
